# Editorial: Parasite, host, and microbiome interactions in natural host systems

**DOI:** 10.3389/fmicb.2025.1589627

**Published:** 2025-04-23

**Authors:** Claire E. Couch, Raquel Xavier, Brianna R. Beechler

**Affiliations:** ^1^U.S. Geological Survey Western Fisheries Research Center, Cook, WA, United States; ^2^CIBIO - Research Centre in Biodiversity and Genetic Resources, Universidade do Porto, Campus de Vairão, Vila do Conde, Portugal; ^3^BIOPOLIS Program in Genomics, Biodiversity and Land Planning, CIBIO, Campus Vairão, Vairão, Portugal; ^4^Department of Biomedical Sciences, Oregon State University Carlson College of Veterinary Medicine, Corvallis, OR, United States

**Keywords:** high-throughput sequencing, parasitology, microbial ecology, host microbe interactions, parasite microbiome interactions

Over the past few decades, high-throughput sequencing (HTS) has evolved from an emerging technology to a central tool for biological research. Advances in HTS technology and computing capacity have reduced costs and improved accessibility, meaning that these tools are now accessible to many disciplines. HTS applications, including but not limited to metagenomics, transcriptomics, and metabarcoding, now allow for the description of gene expression, microbiomes, and parasite communities in a plethora of non-model organisms and ecosystems. The aim of this Research Topic is to highlight the use of HTS to describe parasite communities within natural host populations and to identify and characterize interactions within parasite-microbiome-host systems. We have also included studies on related topics using methods relevant to the characterization of parasite-host-microbiome interactions.

Microbiome studies can reveal potential disease risks in natural host populations. For example, Zhang H. et al. evaluated the presence and abundance of skin microbiota that protect against *Batrachochytrium dendrobatidis* (Bd) in two species of frogs in the Qinling Mountains of China. They also evaluated changes in microbiome communities over time and found greater differences in alpha diversity, beta diversity, and anti-Bd function between seasons than between host species. Their findings highlight the potential importance of seasonal variation in determining infection risk. Nene et al. used 16S and metagenomic sequencing to describe differences and similarities in the microbiome communities of village chickens (*Gallus gallus domesticus*) in two provinces of South Africa. The authors identified several potentially pathogenic taxa in their dataset (e.g., *Escherichia coli* and *Shigella dysenteriae*) in addition to several antimicrobial resistance genes in each province, thus drawing attention to the potential public health implications of free-range chickens.

Host and microbial genetics can be highly informative for understanding the mechanisms of host-microbiome-parasite interactions. Jia et al. used HTS to study the mechanisms of potential biological control agents for the nematode *Bursaphelenchus xylophilus*, a destructive forest pest that causes pine wilt disease. In this study, nematode-trapping fungi were screened for nematocidal efficiency, and mechanisms of efficiency were explored using transcriptome data. They identified a set of gene transcripts related to trap formation and found that both gene expression levels and trap formation were temperature-dependent. Zou et al. used a suite of “omics” methods to understand the functional co-development of yellowfin tuna (*Thunnus albacares*) and their gut microbiomes. They compared gut microbiome composition, metabolites, and mRNA expression in the intestine between juvenile and adult fish, seeking insights into the digestion and metabolism of these endothermic, high-speed swimming fish. They found that adults had higher microbial diversity, gut microbiota-derived metabolites, and intestinal fatty acid production. They also found enriched intestinal gene expression of pathways involved in lipid metabolism in adults. However, juveniles were enriched for protein digestion and absorption, suggesting differences in preferred energy sources between adults and juveniles. It is not always necessary to use HTS for these discoveries; for example, Jackson et al. used Sanger sequencing to investigate how a strain of *Enterococcus faecalis* persists in the GI tract of an invertebrate host, the corn earworm moth (*Helicoverpa zea*). They found that biofilm- and pilus-associated sortase genes are essential for *E. faecalis* biofilm formation, and mutations in these genes lead to reduced persistence in the invertebrate host.

Parasite-microbiome interactions can include interactions between parasites and host microbiomes or between parasites and their own microbiomes. Marsh et al. used 16S sequencing to characterize gut bacteriome diversity and morphological identification of gut parasites to evaluate parasite-gut microbiome interactions in wild wood mice (*Apodemus sylvaticus*). In this study, longitudinal sampling and lethal sampling were conducted to understand both gut ecological trends over time and potential spatial and functional patterns within the gut. In the lethal sampling study, the authors found associations between infection with a specific parasite and microbiome richness in some gut sections; however, results from the non-invasive longitudinal study indicated that microbiome richness was not strongly associated with a single parasite, but it was negatively associated with the total number of parasite species found in the gut. This study highlights how different sampling methodologies can yield different insights into host-microbiome-parasite relationships. Huang et al. evaluated the microbiome of the small hive beetle (*Aethina tumida*, SHB), an invasive pest of honeybees. They found that the SHB gut microbiome shifts to accommodate the change in diet of the beetle from plant fruits outside the hive to bee pollen, honey, and larvae inside the hive. Interestingly, they found that a core honeybee symbiont, *Snodgrassella alvi*, colonized the SHB gut. These findings underscore the adaptability of the parasite-associated microbiome and suggest that the microbiome may play a role in facilitating parasitism. Zhang B. et al. similarly assessed the gut microbiome (bacteria and fungi) of an insect plant pest, *Rhoptroceros cyatheae*, feeding on the leaves of two different plant species. The authors found that the diet significantly altered the gut bacterial diversity, but not the fungal diversity, of *R. cyatheae*, and they found some shared taxa between the insect gut microbiome and the plant host endosymbiont communities. Overall, this study highlights the potential importance of the gut microbiome for adaptation to new hosts.

In conclusion, HTS offers a promising tool for exploring host-parasite-microbiome dynamics at both the ecological level (species interactions) and the functional level (molecular interactions) in natural systems. By taking advantage of high-throughput molecular methods, ecologists can gain new insights into cryptic species interactions. Careful consideration of study design can improve the utility of HTS data, and where possible, multiple sampling methodologies (e.g., non-invasive longitudinal and detailed cross-sectional sampling) could be considered for improved interpretation of results.

